# Supplementary material for: Functional gradients of the cerebellum
Source: eLife. 2018 Aug 14;7:e36652. doi: 10.7554/eLife.36652 (PMC6092123; doi:10.7554/eLife.36652)
Supplement: Supplementary file 1. [file elife-36652-supp1.docx]

|  | **Task activity maps (Guell et al., 2018)** | **Resting-state network maps (Buckner et al., 2011)** |
| --- | --- | --- |
| **Participants** | - 787 participants; 431 female; ages 22-25 (n=172), 26-30 (n=337), 31-35 (n=272), >35 (n=6)  - Exclusion criteria: diabetes or high-blood pressure (for neuroimaging data quality purposes), neurodevelopmental, neuropsychiatric or neurological disorders, and genetic disorders. | - 1,000 participants; 537 female; ages 18-35  - Exclusion criteria: self-reported health information indicating a history of neurological or psychiatric illness, or taking psychoactive medications. |
| **Scanner parameters** | - Customized 3T Siemens Skyra; 32-channel head coil  - Structural scan (T1w) repetition time / inversion time / echo time / flip angle / voxel resolution / field of view: 2,400ms / 1000ms / 2.14ms / 8° / 0.7mm isotropic / 224mm  - Functional scan repetition time / echo time / flip angle / voxel resolution / field of view / number of slices: 720ms (multiband factor = 8) / 33 ms / 52° / 2mm isotropic / 208mm / 72 slices | - 3T Tim Trio scanner (Siemens); 12-channel head coil  - Structural scan (T1w) repetition time / inversion time / echo time / flip angle / voxel resolution / field of view: 2,200ms / 1,100ms / from 1,54 ms to 7.01 ms / 4° / 1.2mm isotropic / 230mm  - Functional scan repetition time / echo time / flip angle / voxel resolution / field of view / number of slices: 3,000ms / 30ms / 85° / 3mm isotropic / 216mm / 47 slices. 124 data points were acquired. |
| **Conditions** | - Motor: movement (tap left fingers, or tap right fingers, or squeeze right toes, or squeeze left toes, or move tongue) minus Average (average of the other four movements)  - Working memory: Two back (subject responds if current stimulus matches the item two back) minus Zero back (subject responds if current stimulus matches target cue presented at start of block)  - Language: Story (listen to stories) minus Math (answer arithmetic questions)  - Social: TOM (view socially interacting geometric objects) minus Random (view randomly moving geometric objects)  - Emotion: Faces (decide which of two angry/fearful faces on the bottom of the screen match the face at the top of the screen) minus Shapes (same task performed with shapes instead of faces) | - Rest: participants were instructed to remain still, stay awake, and keep their eyes open. |
| **Functional data preprocessing** | - Slice timing correction: not necessary given short repetition time  - Motion correction: head motion correction using 6DOF realignment, motion correction parameters were included as nuisance regressors  - Registration: cerebral cortical surface registered using Freesurfer’s Boundary-Based Registration^1^; cerebellum registered using nonlinear volumetric registration.  - Smoothing: cerebellar smoothing was performed with 2mm FWHM constrained to the cerebellum to avoid mixing signals from adjacent brain structures.  - Other relevant corrections: correction of gradient-nonlinearity-induced distortion (important in HCP custom 3T scanner). | - First four runs were discarded to allow for T1 equilibration  - Slice timing correction  - Motion correction: head motion correction using 6DOF realignment, motion correction parameters were included as nuisance regressors  - Registration: cerebral cortical surface registered using Freesurfer’s Boundary-Based Registration^1^, cerebellum registered using nonlinear volumetric registration.  - Smoothing: cerebral cortical surface and cerebellar volume smoothing was performed with 6mm FWHM. Cerebellar smoothing with 6mm FWHM was constrained to the cerebellum to avoid mixing signals from adjacent brain structures.  - Other relevant corrections: low pass temporal filtering, whole brain signal regression, and ventricular and white matter signal regression: this minimized nonneural signal correlations, but decreased the interpretability of negative correlations. Accordingly, further analyses included positive correlations only. Signal from cerebral cortex immediately adjacent to the cerebellum was regressed from the cerebellar signal. |
| **Analysis** | - Individual subject contrast of parameter estimates (cope) were generated using FSL’s FEAT General Linear Model contrasting experimental and control conditions in a blocked design  - Cope files from each individual subject were transformed to a Cohen’s d group map by concatenating all individual cope files, extracting the mean and standard deviation from this concatenated file, and calculating (cope mean / cope SD).  - A Cohen’s d threshold of 0.5 (medium effect size) was used to generate the task activity maps. | - Correlation maps of individual subjects were converted to z-maps using Fisher’s r-to-z transform. An inverse Fisher’s r-to-z transformation was applied to the group-averaged correlation z-map, generating a group-averaged correlation map  - 1,175 regions of interest were defined in the cerebral cortex (uniformly sampled in surface space, consisting of single vertices spaced 16mm apart). These regions of interest were correlated with all cerebral cortical surface vertices (18,715), top 10% correlations were retained, and a clustering algorithm^2^ was applied to this 18,715x1,175 matrix. This clustering approach generated a map of 7 cerebral cortical networks.  - Connectivity between each cerebellar voxel and these 1,175 cortical regions of interest was calculated. A winner-takes-all algorithm defined which of the 7 cerebral cortical networks had the strongest connectivity for each cerebellar voxel, generating a cerebellar resting-state networks map. |

**Supplementary File 1**. Methodological details of task activity^3,4^ and resting-state network^5,6^ maps.

1. Greve, D. N. & Fischl, B. Accurate and robust brain image alignment using boundary-based registration. *Neuroimage* **48,** 63–72 (2009).

2. Lashkari, D., Vul, E., Kanwisher, N. & Golland, P. Discovering structure in the space of fMRI selectivity profiles. *Neuroimage* **50,** 1085–1098 (2010).

3. Guell, X., Gabrieli, J. DE & Schmahmann, J. D. Triple representation of language, working memory, social and emotion processing in the cerebellum: Convergent evidence from task and seed-based resting-state fMRI analyses in a single large cohort. *Neuroimage* **172,** 437–449 (2018).

4. Glasser, M. F. *et al.* The minimal preprocessing pipelines for the Human Connectome Project. *Neuroimage* **80,** 105–124 (2013).

5. Buckner, R. L., Krienen, F. M., Castellanos, A., Diaz, J. C. & Yeo, B. T. T. The organization of the human cerebellum estimated by intrinsic functional connectivity. *J. Neurophysiol.* **106,** 2322–45 (2011).

6. Yeo, B. *et al.* The organization of the human cerebral cortex estimated by intrinsic functional connectivity. *J. Neurophysiol.* **106,** 1125–1165 (2011).
